# Autophagy deficiency exacerbates colitis through excessive oxidative stress and MAPK signaling pathway activation

**DOI:** 10.1371/journal.pone.0225066

**Published:** 2019-11-08

**Authors:** Minori Kubota, Kazuki Kakimoto, Takatoshi Nakagawa, Eiko Koubayashi, Kei Nakazawa, Hideki Tawa, Yuki Hirata, Toshihiko Okada, Ken Kawakami, Akira Asai, Shuhei Hosomi, Toshihisa Takeuchi, Shinya Fukunishi, Takuya Inoue, Michio Asahi, Kazuhide Higuchi

**Affiliations:** 1 2nd Department of Internal Medicine, Osaka Medical College, Daigakumachi, Takatsuki, Osaka, Japan; 2 Department of Pharmacology, Faculty of Medicine, Osaka Medical College, Daigakumachi, Takatsuki, Osaka, Japan; 3 Department of Gastroenterology, Osaka City University Graduate School of Medicine, Asahimachi, Abeno-ku, Osaka, Japan; University of the Pacific, UNITED STATES

## Abstract

**Background and aim:**

Autophagy is an essential process involved in the pathogenesis of inflammatory bowel disease (IBD). Although there are many data showing the roles of autophagy in intestinal epithelial cells (IECs), the mechanisms involved remain to be fully elucidated. We investigated the influence of autophagy in IECs on gastrointestinal tract inflammation.

**Methods:**

Mice with conditional knockout of *Atg5* in IECs (*Atg*5^flox/flox^/villin-Cre mice) were subjected to dextran sulfate sodium (DSS)-induced colitis and analyzed for colitis susceptibility. Additionally, we used Atg5-silenced rat IECs (IEC6shAtg5 cells) for *in vitro* assays.

**Results:**

Sensitivity to DSS markedly increased in *Atg*5^flox/flox^/villin-Cre mice compared to that in wild-type mice. In IEC6shAtg5 cells, apoptosis was enhanced, and cell viability significantly decreased compared to IEC-6 cells. The expression of proinflammatory cytokines increased upon suppression of autophagy. Furthermore, silencing of *Atg5* was associated with inflammation of IECs, activation of the mitogen-activated protein kinase (MAPK) signaling pathway by the intracellular reactive oxygen species accumulation, and NF-κB p65 phosphorylation.

**Conclusions:**

Autophagy in IECs plays an essential role in the maintenance of intestinal homeostasis, and autophagy deficiency triggers inflammation. Development of methods targeting autophagy might be beneficial in the treatment of IBD.

## Introduction

Inflammatory bowel disease (IBD), which includes Crohn’s disease and ulcerative colitis (UC), is an intractable condition that causes chronic inflammation in the mucosa of the small and large intestine. Numerous studies have attempted to characterize the pathogenic mechanism of IBD. Interestingly, genome-wide association studies (GWAS) have identified the autophagy-related 16-like 1 (*ATG16L1*) and immunity-related GTPase family M (*IRGM)* genes, as genes associated with susceptibility to Crohn’s disease [1–3].

Analysis using autophagy-related factors in genetically-modified mice revealed that autophagy in macrophage is essential for maintaining homeostasis and innate immunity physiology[4, 5]. Conversely, autophagy in IECs is critical for maintaining intestinal homeostasis [4, 6, 7]. Paneth cells are epithelial cells found in the crypts of the small intestine, which store and secrete antimicrobial peptides such as defensins, contained in cytoplasmic granules[8]. Autophagy is vital for maintaining the function of Paneth cells: in mice with reduced expression of Atg16L1, the antimicrobial function mentioned above is diminished due to a failure of Paneth cell granules to form, and a similar phenomenon is observed in patients with Crohn’s disease carrying homozygotic risk alleles of *ATG16L1*[8].

Given that gastrointestinal inflammation has been observed in other parts of the gastrointestinal tract other than the small intestine in patients with Crohn’s disease, inflammation due to autophagy deficiency may not depend on only the function of Paneth cells. We previously reported that autophagy deficiency in IECs increased reactive oxygen species (ROS) production in *Atg*5^flox/flox^/villin-Cre mice and IEC6shAtg5 cells [9]. This study aimed to clarify the relationship between autophagy in IECs and gastrointestinal inflammation using *Atg5*^flox/flox^/villin-Cre mice and IEC6shAtg5 cells.

## Materials and methods

### Reagents

Bafilomycin (Cat. No. 88899-55-2) and MEK inhibitor (PD98059) (Cat. No. 1213) were purchased from Wako Pure Chemical Inc. (Osaka, Japan). Lipopolysaccharides (Cat. No. L4130) and N-Acetyl-L-Cysteine (NAC) (Cat. No. 9165) were purchased from Sigma-Aldrich (St. Louis, MO). 3-methyladenine (3-MA) (Cat. No. SC-205596) was purchased from Santa Cruz (La Jolla, CA). NF-κB transcriptional activity inhibitor (JSH23) (Cat. No. ab144824) was purchased from Abcam (Cambridge, UK).

### Mice

Villin-Cre mice and mice with a floxed *Atg5* allele were purchased from the Jackson Laboratory (Bar Harbor, ME) and RIKEN BioResource Center (Saitama, Japan), respectively. Male *Atg5*^flox/flox^/villin-Cre mice, which were IEC-specific *Atg5* conditional knockout mice, were generated by crossing *Atg5*^flox/+^ mice and villin-Cre-expressing mice as described previously[9].

### Animal models of colitis

To create colitis model mice (C57BL/6), wild-type (WT), *Atg5*^flox/+^/villin-Cre, and *Atg5*^flox/flox^/villin-Cre mice, all aged 8–10 weeks, were given 2.5% DSS (Nacalai Tesque, Kyoto, Japan) in drinking water for six days, and plain drinking water afterward. The body weights of the mice were measured every day, and, on day eight after DSS administration, the mice were sacrificed. The histological scores of the Hematoxylin and eosin (HE)-stained rectal specimens were determined as described previously[10]. Ethical permission for the experiments presented in this study was given by the Animal Ethical Committee of the Osaka Medical College, and all procedures were conducted according to the guidelines of the Institute for Laboratory Animal Research at Osaka Medical College (permission # 30049).

### Cell culture and development of an Atg5 knockdown cell line

IEC-6 cells, which are rat IECs, were obtained from RIKEN BioResource Center (Saitama, Japan) and cultured in Dulbecco’s modified Eagle’s medium containing 10% fetal bovine serum, 100 U/mL penicillin, and 100 mg/mL streptomycin. Atg5 knockdown IEC-6 cells (IEC6shAtg5 cells) were generated as described previously[9]. IEC6shAtg5 cells were cultured in IEC-6 growth medium with 1 mg/mL puromycin.

### Quantitative polymerase chain reaction (qPCR)

To measure the expression levels of the proinflammatory cytokines IL-1β, IL-6, and tumor necrosis factor (TNF)-α, qPCR was performed as described previously[11]. Briefly, total RNA was extracted using a total RNeasy mini-Kit (Qiagen, Hulsterweg, Netherlands). For cDNA synthesis, RNA was reverse-transcribed with a Prime Script RT reagent Kit (Takara Bio Inc., Shiga, Japan) with a SYBR Premix Ex Taq Kit (Takara Bio Inc.) on the Thermal Cycler Dice Real Time System TP870 (Takara Bio Inc.). The sequences of sense and antisense primers for mouse *TNF-α*, *IL-1β*, *IL-6*, and *GAPDH* are depicted in Table 1.

**Table 1:** The sequences of sense and antisense primers used.

Gene	Species	Primer Sequence
*IL-1β*	mouse	F: 5'-GCTATGGCAACTGTTCCT R: 5'-AAGCAGCCCTTCATCTTT
*IL-6*	mouse	F: 5'-TTCCTCACTGTGGTCAGA R: 5'-CATTCATATTGTCAGTTCTTCGTA
*TNF-α*	mouse	F: 5'-ACCTTGTTGCCTCCTCTT R: 5'-GTTCAGTGATGTAGCGACAG
*GAPDH*	mouse	F: 5'-CGACTTCAACAGCAACTC R: 5'-TATTCATTGTCATACCAGGAA
*IL-1β*	rat	F: 5'-CACCTCTCAAGCAGAGCACAG R: 5'-GGGTTCCATGGTGAAGTCAAC
*IL-6*	rat	F: 5'-TCCTACCCCAACTTCCAATGCTC R: 5'-TTGGATGGTCTTGGTCCTTAGCC
*GAPDH*	rat	F: 5'-GTATTGGGCGCCTGGTCACC R: 5'-CGCTCCTGGAAGATGGTGATGG

### Western blot analysis

Western blot analysis was performed as described previously[9]. Briefly, cultured cells were homogenized in a lysis buffer containing a protease inhibitor cocktail. After SDS-PAGE, the proteins on the gel were transferred to a polyvinylidene membrane. Then, the membrane was stained with the desired antibodies. The membrane was visualized with ImmobilonWestern (Millipore, Hayward, CA), and the image was captured with ChemDoc XRS (Bio-Rad, Hercules, CA). Primary antibodies used for Western blotting: microtubule-associated protein 1 light chain 3 (LC3) rabbit polyclonal antibody (PM036; MBL, Nagoya, Japan), p62 mouse monoclonal antibody (ab56416; Abcam, Cambridge, UK), PARP1 rabbit polyclonal antibody (9542S; Cell Signaling Technology, Danvers, MA), phospho-NF-kB p65 rabbit monoclonal antibody (Ser536) (3033S; Cell Signaling Technology), NF-kB p65 rabbit polyclonal antibody (sc-372; Santa Cruz Biotech, Dallas, TX), phosphor-extracellular signal–regulated kinase (ERK) rabbit polyclonal antibody (9101S; Cell Signaling Technology), ERK rabbit monoclonal antibody (4695S; Cell Signaling Technology), tubulin rabbit monoclonal antibody (ab176560; Abcam). Goat anti-rabbit IgG-HRP (sc-2030; Santa Cruz) or goat anti-mouse IgG-HRP (sc-2005; Santa Cruz) were used as secondary antibodies.

### Evaluation of cell viability

Cells were plated in 96-well plates and incubated for 24 h with or without hydrogen peroxide (H_2_O_2_). The cells were next incubated with Cell Counting Kit-8 (Wako Pure Chemical Industries, Osaka, Japan) for 1 h, and the absorbance in each well was measured at a wavelength of 450 nm using a microplate reader (model 680; Bio-Rad). Inhibition of viability was determined relative to IEC-6 cells without H_2_O_2._treatment.

### Evaluation of oxidative stress

The Total ROS Detection Kit (Enzo Life Sciences Inc., New York, USA) was used to evaluate the levels of oxidative stress in IEC-6 and IEC6shAtg5 cells[9] before and after H_2_O_2_ treatment. Fluorescence was directly measured with a GloMax Multi-Detection System.

### Statistical analysis

Statistical significance was evaluated using the Student’s *t*-test for parametric data, which are presented as mean ± the standard deviation (SD). Non-parametric data (histological evaluations) were analyzed using the Mann-Whitney test. Statistical significance was defined as *p* < 0.05.

## Results

### Atg5 knockout worsens colitis in mice

**Fig 1:**
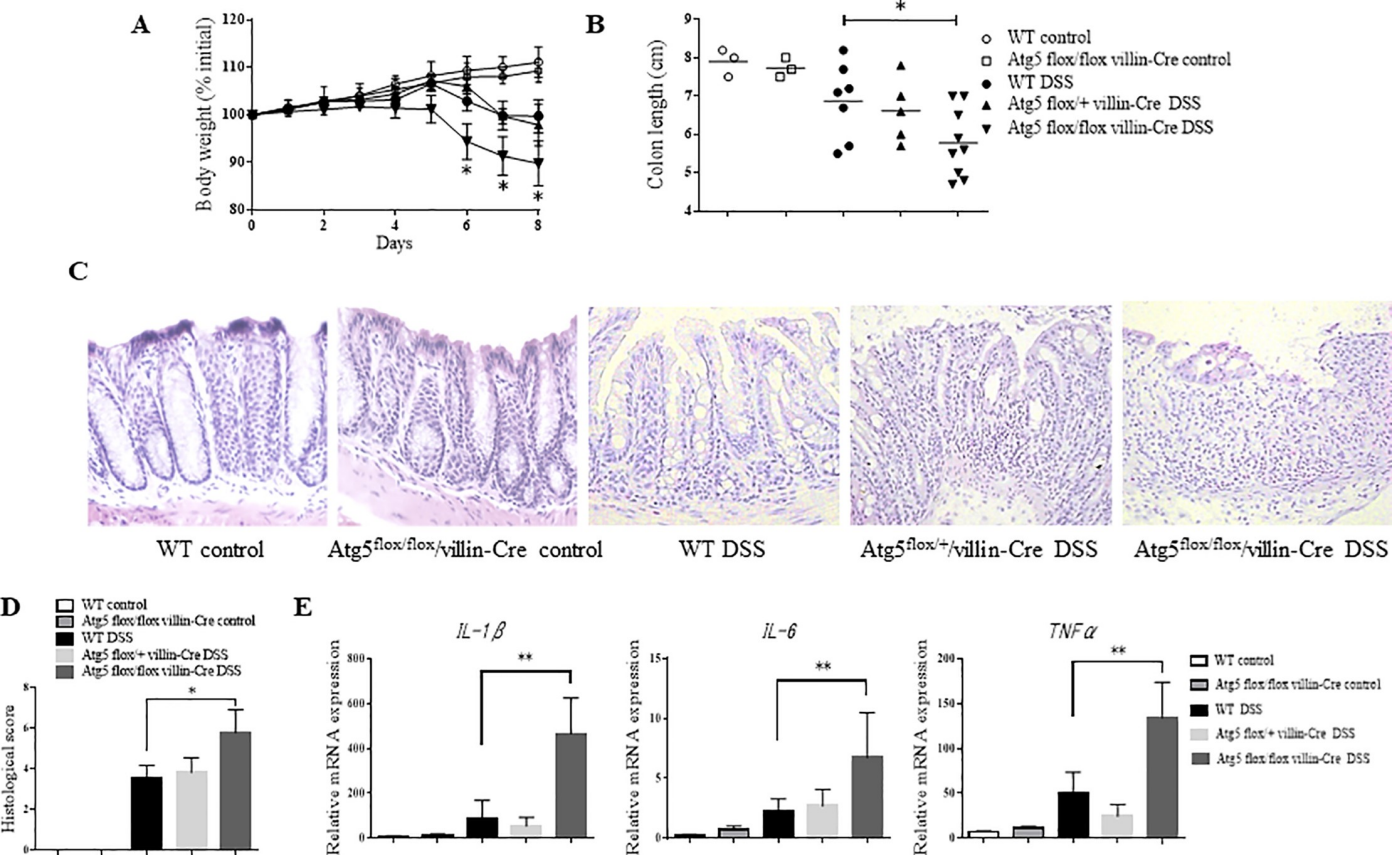
Atg5flox/flox/villin-Cre mice are more sensitive to DSS-induced colitis than WT mice. (A) Acute colitis was induced in WT, Atg5flox/+/villin-Cre, and Atg5flox/flox/villin-Cre mice by oral feeding of DSS (n = 5–9 per group). Weight loss during colitis progression is shown. Mice with colitis were euthanized on day 8, and severity of DSS-induced colitis was determined by colon length (B) and histological score (D). (C) Histological changes in the middle segment of the colon. (E) mRNA expression of proinflammatory cytokine genes in colon tissues quantified by qPCR. Data are expressed as the relative abundance of GAPDH. Results are expressed as the mean ± SD; *p < 0.05, **p < 0.01.

To investigate the effect of *Atg5* deficiency in IECs on colitis, we prepared DSS colitis models using WT, *Atg*5^flox/+^/villin-Cre, and *Atg5*^flox/flox^/villin-Cre mice. *Atg5*^flox/+^/villin-Cre mice exhibited the same weight loss as WT mice, but the weight loss in *Atg5*^flox/flox^/villin-Cre mice was significantly lower than that in WT mice (Fig 1A). Additionally, the intestinal length was significantly shortened in *Atg5*^flox/flox^/villin-Cre mice compared with controls (Fig 1B). Fig 1C displays typical photographs representative of the colonic tissues of each mouse group. In tissues from WT and Atg5^flox/flox^/villin-Cre mice (control), no epithelial lesions were observed. Although loss of crypts and inflammatory cells infiltration to the lamina propria were observed in all mice after DSS administration, the severity was higher in Atg5^flox/flox^/villin-Cre mice than in WT mice. We next evaluated the histological scores indicating the extent of inflammation in the intestinal tissues. The histological score was significantly higher in *Atg5*^flox/flox^/villin-Cre mice (5.76±1.15) than in the WT (3.53±0.64) and *Atg5*^flox/+^/villin-Cre mice (3.81±0.72) (Fig 1D). Next, we analyzed the expression of proinflammatory cytokines in colon tissues by qPCR, and, as a result of DSS administration, we found that the expression of *IL-1β*, *IL-6*, and *TNF-α* was significantly increased in *Atg5*^flox/flox^/villin-Cre mice (Fig 1E). Conversely, *Atg5*^flox/+^/villin-Cre and WT mice exhibited no difference in the expression of proinflammatory cytokines. Overall, these data indicated that inhibition of autophagy through *Atg5* knockout in IECs exacerbates colitis.

### *Atg5*-silencing reduces cell viability and induces apoptosis

**Fig 2:**
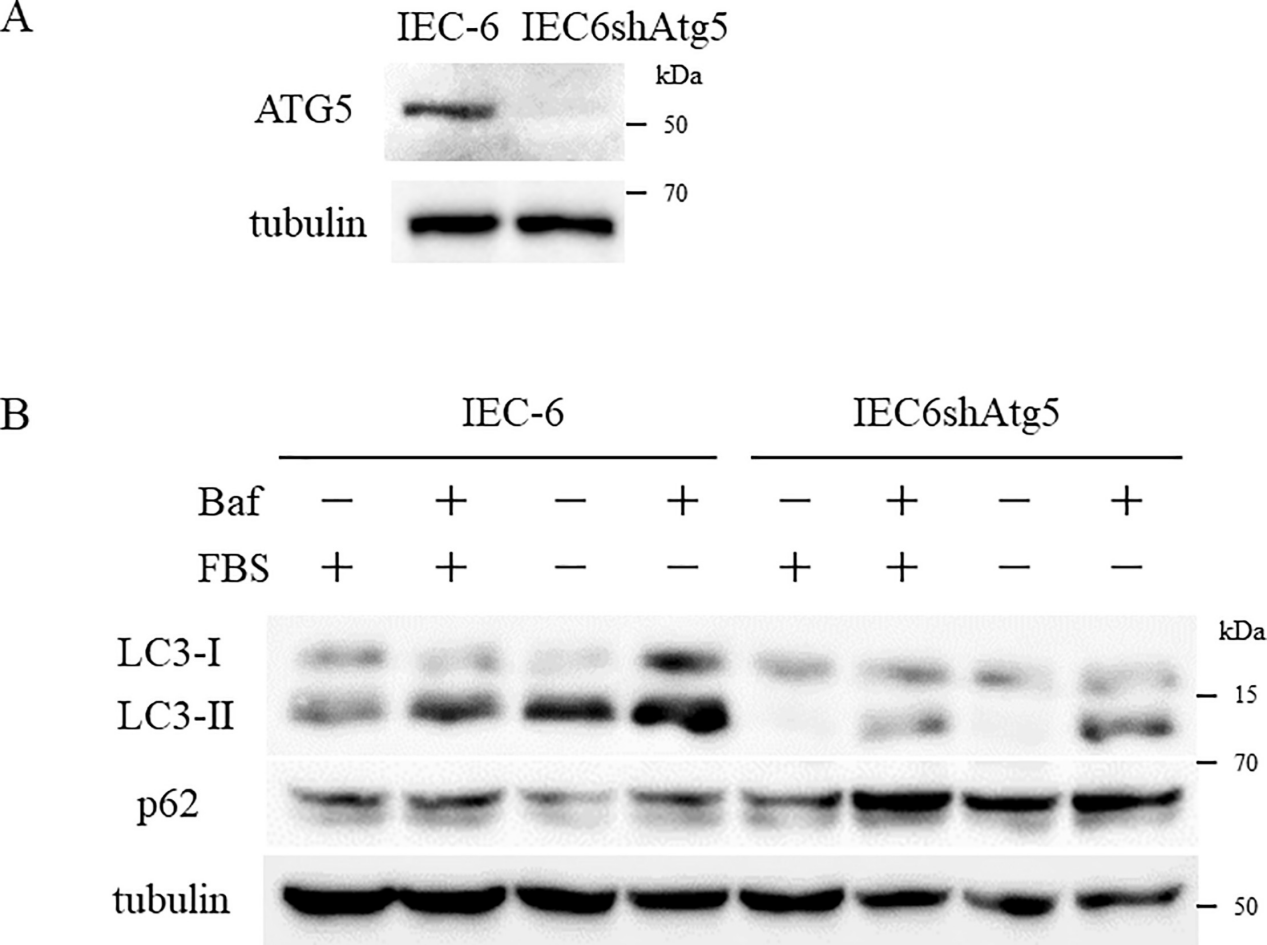
The autophagic flux is normal in IEC-6 cells but atg5-silencing inhibits autophagy. (A) LC3 expression levels in IEC-6 and IEC6shAtg5 cells were determined using Western blot analysis. IEC-6 cells were cultured in a standard culture medium or in a serum-free culture medium for 24 h. (B) LC3 expression levels in IEC-6 cells with or without Bafilomycin A1 (Baf) for 24 h were determined to confirm the presence of autophagic flux.

To further investigate the role of autophagy in IECs, we exploited IEC6shAtg5 cells. As we reported previously, autophagy in the cells was almost completely suppressed judging by the amount of the lipidated form of LC3, LC3-II[9]. To confirm the capability of IEC-6 cells to engage in autophagy, the cells were cultured in a serum-free culture medium for 24 h. We found that LC3-II expression was significantly enhanced in the cells cultured in a serum-free culture medium compared to the ones in the standard culture medium (Fig 2). LC3-II was rarely detected in IEC6shAtg5 cells in both culture mediums. To confirm whether the expected autophagic flux was occurring, we incubated IEC-6 cells with Bafilomycin A1 (Baf), a lysosome inhibitor, for 24 h and found that LC3-II expression increased markedly, indicating that the autophagic flux in IEC-6 cells was in fact occurring (Fig 2). A similar result was obtained when we repeated the experiments with cells cultured in serum-free medium for 24 h. Furthermore, to convince the autophagy suppression in IEC6shAtg5 cells, we examined p62 expression level, which is an autophagosome cargo protein, in these cells with Western blot analysis, and found that p62 expression was enhanced in IEC6shAtg5 cells compared with in IEC-6 cells (Fig 2). These results indicate that the autophagic flux occurred in IEC-6 cells but was almost entirely suppressed in IEC6shAtg5 cells.

**Fig 3:**
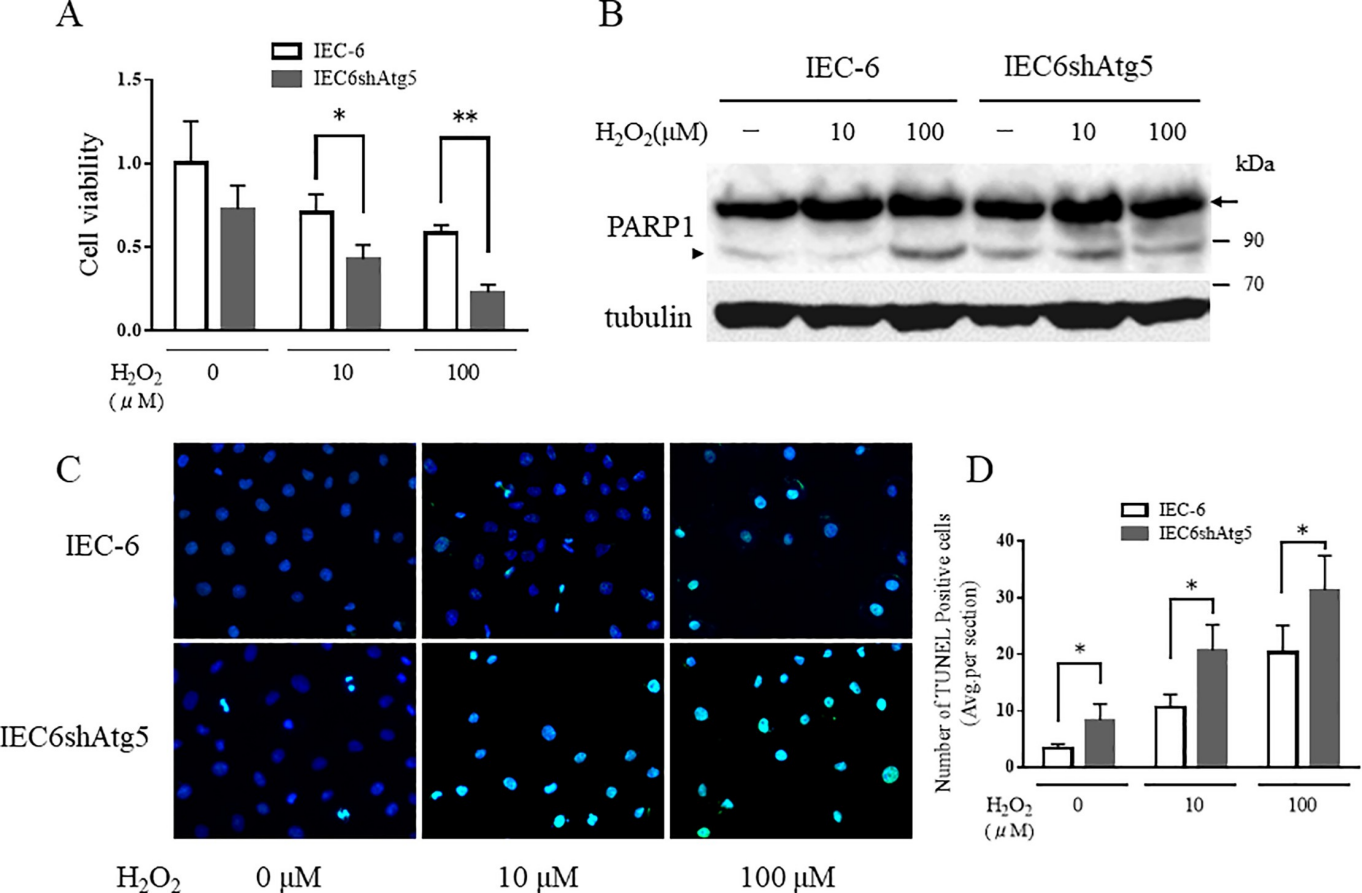
Atg5-silencing reduces cell viability and induces apoptosis in IEC-6 cells. (A) The relative cell viability in IEC-6 and IEC6shAtg5 cells with or without H_2_O_2_ treatment. These cells were treated with 0, 10, and 100 μM H_2_O_2_ for 24 h. Statistical analysis was performed using Tukey’s method. *p < 0.05, **p < 0.01. (B) Intact and cleaved PARP1 expression levels in IEC-6 and IEC6shAtg5 cells treated with 0, 10, and 100 μM H_2_O_2_ for 24 h. The arrow and arrowhead indicate intact and cleaved PARP1, respectively. (C) TUNEL assay in IEC-6 and IEC6shAtg5 cells treated with 0, 10, and 100 μM H_2_O_2_ for 24 h.

To investigate the influence of suppression of autophagy on cell viability of IECs, IEC-6 and IEC6shAtg5 cells were exposed to H_2_O_2_. As we previously reported that IEC6shAtg5 cells produced higher ROS than IEC-6 cells did [9], we used H_2_O_2_ as an inducer of oxidative stress. Although the viability of IEC6shAtg5 cells was lower than that of IEC6 cells before H_2_O_2_ exposure, the difference was not significant. In contrast, the difference became significant upon H_2_O_2_ exposure (Fig 3A). Next, to confirm whether apoptosis was involved in the decline of cell viability, we examined the cleavage of PARP1, which is a substrate of caspase-3, by Western blot analysis, and found that PARP1 cleavage was significantly enhanced in IEC6shAtg5 cells compared to that in IEC-6 cells, especially at a low H_2_O_2_ concentration (10 μM) (Fig 3B). PARP1 cleavage was almost the same in both cells at a higher amount of H_2_O_2_ (100 μM). Furthermore, we also performed TUNEL assay to confirm that the susceptibility to oxidative stress might be due to apoptosis. The result showed that the number of TUNEL positive cells was significantly higher in IEC6shAtg5 cells compared with in IEC-6 cell (Fig 3C and 3D). These findings suggest that suppression of autophagy lowers cell viability by the induction of apoptosis through oxidative stress.

### Inhibition of autophagy induces the expression of proinflammatory cytokines and the phosphorylation of NF-κB

**Fig 4:**
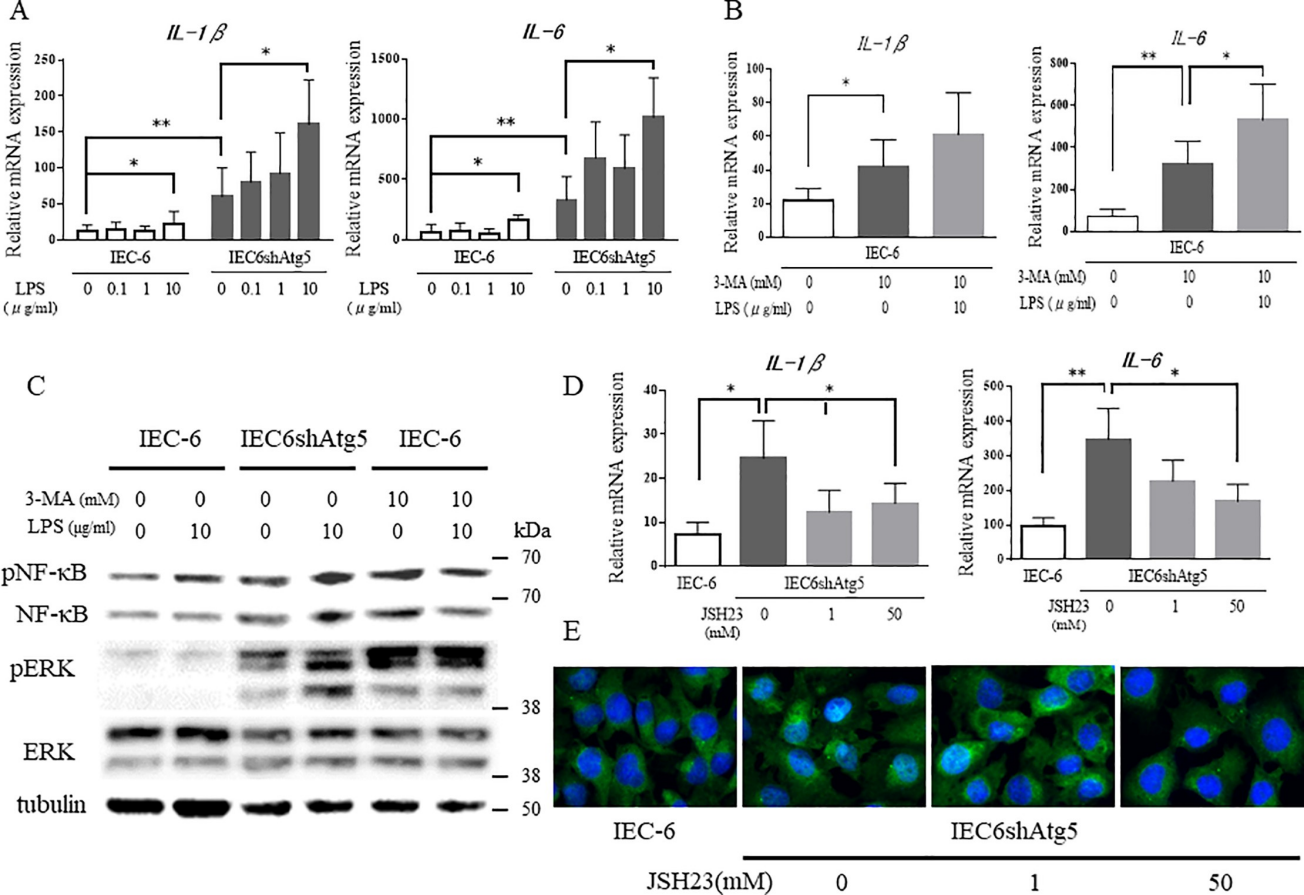
Inhibition of autophagy induces the expression of proinflammatory cytokine genes and the phosphorylation of NF-κB. (A) mRNA expression of proinflammatory cytokine genes in IEC-6 and IEC6shAtg5 cells after LPS treatment. Cells were treated with 0, 0.1, 1, and 10 μg/ml LPS for 24 h, and mRNA expression of IL-1β and IL-6 were determined by qPCR. (B) mRNA expression of proinflammatory cytokine genes in IEC-6 cells with or without LPS or 3-MA treatment. Cells were treated with or without LPS (10 μg/ml) or 3-MA (10 mM) treatment for 24 h, and mRNA expression of IL-1β and IL-6 were determined by qPCR. (C) pNF-κB p65 and pERK protein expression levels in IEC-6 and IEC6shAtg5 cells with or without LPS or 3-MA treatment. Cells were treated with or without LPS (10μg/ml) or 3-MA (10 mM) for 24 h, and pNF-κB p65 and pERK protein expression levels were determined by Western blot analyses. Experiments were replicated at least three times, and the representative figures are shown. (D) mRNA expression of proinflammatory cytokines in IEC-6 and IEC6shAtg5 cells after NF-κB inhibition. Cells were treated with or without an inhibitor of NF-κB transcriptional activity, JSH23, and mRNA expression of IL-1β and IL-6 was determined by qPCR. Experiments were replicated at least three times. *p < 0.05, **p < 0.01.

To investigate the influence of autophagy in IECs on inflammation, we measured the expression levels of proinflammatory cytokines in IEC-6 and IEC6shAtg5 cells by qPCR. *IL-1β* and *IL-6* mRNA expression levels were markedly elevated in IEC6shAtg5 cells compared to those in IEC-6 cells (Fig 4A). Moreover, when stimulated with LPS, the expression levels increased in both cells in a dose-dependent manner. After stimulation with 10 μg/mL LPS, the expression levels were significantly higher in both cells than in the non-stimulated cells. To evaluate the involvement of autophagy in inflammation via another means, the effect of 3-MA, which suppresses autophagy, on the expression of proinflammatory cytokines in IEC-6 cells was assessed. 3-MA inhibited an increase of LC3-II induced by serum-depletion (-FBS) in IEC-6 cells (S1 Fig). After treatment with 3-MA (10 mM) for 24 h, the expression of *IL-1β* and *IL-6* increased markedly as observed in IEC6shAtg5 cells, confirming that suppression of autophagy in IEC-6 cells genetically or pharmacologically increases the expression of proinflammatory cytokines (Fig 4B).

To investigate the mechanism by which autophagy suppression increased the expression of proinflammatory cytokines, we examined the phosphorylation of NF-κB p65, a master intracellular transcription factor involved in inflammation, by Western blot analysis in IEC-6 and IEC6shAtg5 cells. In IEC6shAtg5 cells, NF-κB p65 phosphorylation was markedly enhanced compared to IEC-6 cells (Fig 4C). NF-κB p65 phosphorylation was similarly enhanced in IEC-6 cells supplemented with 3-MA. These results strongly suggested that the increased expression of proinflammatory cytokines upon autophagy suppression was mediated by NF-κB p65 phosphorylation. Furthermore, to convince the fact that NF-κB is involved in the expression of proinflammatory cytokines in IEC6shAtg5 cells, the effect of JSH23, an inhibitor of NF-κB transcriptional activity, on the expression of proinflammatory cytokines in IEC6shAtg5 cells was assessed. After the treatment with JSH23 for 24 h, the expression of IL-1β and IL-6 decreased markedly to the same level as that in IEC-6 cells, suggesting that the increased expression of proinflammatory cytokines upon autophagy suppression was mediated by NF-κB activation (Fig 4D). Nuclear localization of NF-κB was observed in IEC6shAtg5 cells, not in IEC-6 cells at the basal state, which was blocked by the treatment with 50 μM JSH23 for 24 h (Fig 4E).

### Inhibition of autophagy induces ROS accumulation and MAPK signaling pathway activation

**Fig 5:**
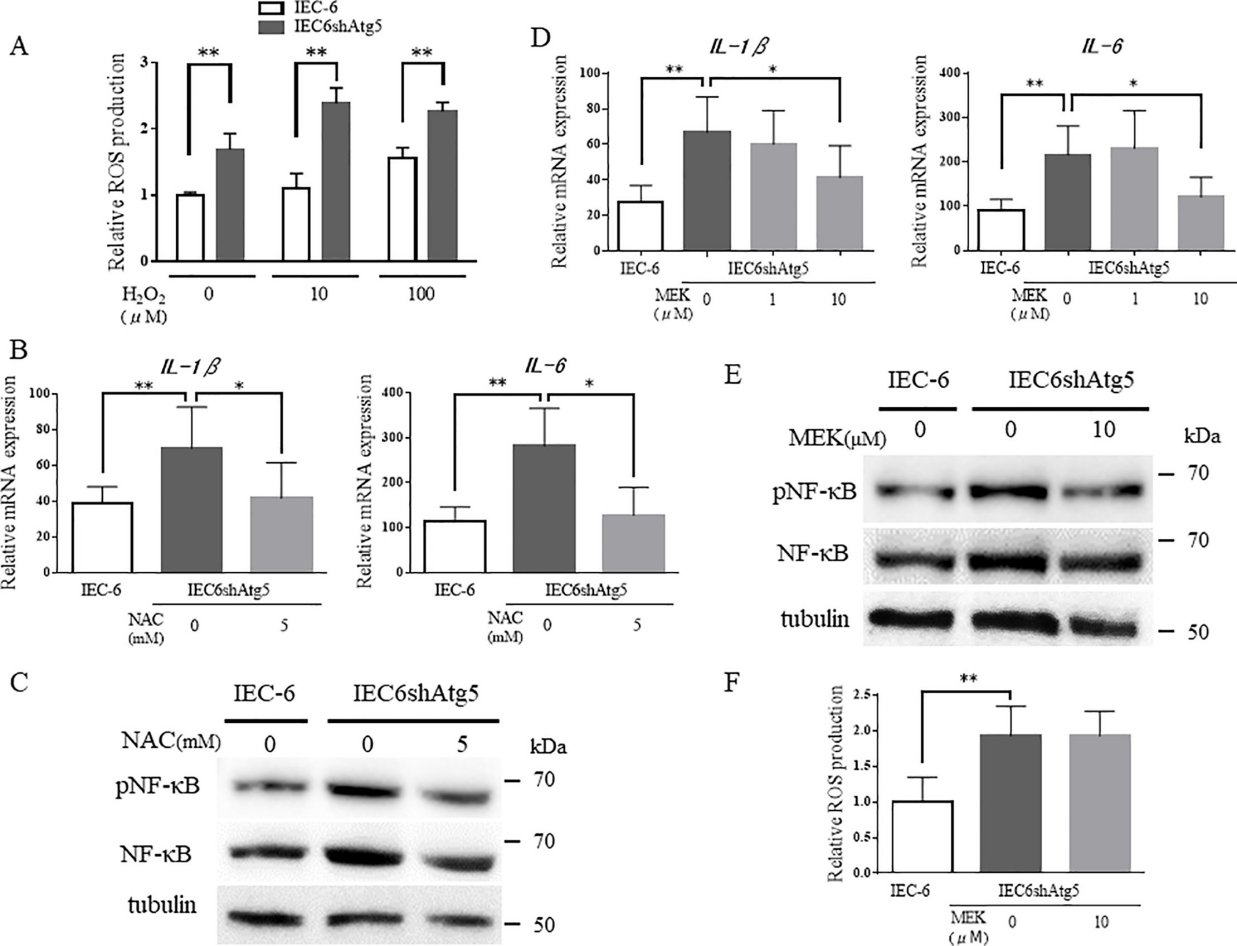
Inhibition of autophagy induces the accumulation of ROS and activation of the MAPK signaling pathway. (A) Total ROS levels in IEC-6 and IEC6shAtg5 cells after H_2_O_2_ treatment. Cells were treated with 0, 10, and 100 μM H_2_O_2_ for 24 h, and total ROS levels were evaluated with the Total ROS Detection Kit (Enzo Life Sciences Inc., NY) (B) mRNA expression of proinflammatory cytokine genes in IEC-6 and IEC6shAtg5 cells after ROS removal. Cells were treated with 0, 1, and 5 mM NAC, and mRNA expression of IL-1β and IL-6 were determined by qPCR. (C) pNF-κB p65 and pERK protein expression levels in IEC6shAtg5 cells after ROS removal. Cells were treated with or without 5 mM NAC for 24 h, and the expression levels were determined by Western blot analyses. (D) mRNA expression levels of proinflammatory cytokines in IEC6shAtg5 cells treated with a MEK inhibitor, PD98059. Cells were treated with 0, 1, 10, and 100 μM PD98059, and mRNA expression of IL-1β and IL-6 were determined by qPCR. *p < 0.05, **p < 0.01. (E) pNF-κB p65 and pERK protein expression levels in IEC-6 and IEC6shAtg5 cells after PD98059 treatment. Cells were treated with or without 10 μM PD98059, and the expression levels were determined by Western blot analyses. (F) Total ROS levels in IEC-6 and IEC6shAtg5 cells with or without PD98059. Cells were treated with 0, 10, 100 μM of a MEK inhibitor, PD98059, and mRNA expression of IL-1β and IL-6 was determined by qPCR. *p < 0.05, **p < 0.01.

To examine the mechanism by which autophagy suppression results in an increased expression of proinflammatory cytokines, we focused on the level of oxidative stress. As we reported previously, the suppression of autophagy in IEC-6 cells triggered the intracellular accumulation of total ROS[9], which was further increased with 10 and 100 μM H_2_O_2_ (Fig 5A). Next, the effect of an ROS scavenger (NAC, 5 mM) on the expression of proinflammatory cytokines was examined in IEC6shAtg5 cells. We found that *IL-1β* and *IL-6* expression significantly decreased (Fig 5B). These results demonstrated that the expression of proinflammatory cytokines is enhanced by the intracellular accumulation of ROS due to autophagy suppression.

The MAPK signaling pathway is activated by oxidative stress and plays an essential role in cellular function. It is also known that the activation of this pathway leads to the activation of the NF-κB pathway as well. Indeed, the phosphorylation of ERK, the MAPK signaling pathway, was upregulated in IEC6shAtg5 cells (Figs 4C and 5C). After the treatment with 3-MA, which suppresses autophagy, the phosphorylation was also upregulated in IEC-6 cells (Fig 4C). Therefore, we hypothesized that the ERK upregulation might be involved in the increase in proinflammatory cytokines observed in IEC6shAtg5 cells compared to IEC-6 cells. The upregulation of ERK in IEC6shAtg5 cells was reversed by NAC (Fig 5C). Inhibition of the MAPK signaling pathway with a MEK inhibitor, PD98059, resulted in the significant suppression of *IL-1β* and *IL-6* expression in IEC6shAtg5 cells (Fig 5D). PD98059 also suppressed NF-κB p65 phosphorylation (Fig 5E), whereas it did not suppress ROS production (Fig 5F). These data indicate that the intracellular accumulation of ROS, caused by autophagy suppression, increases the expression of proinflammatory cytokines via the MAPK and NF-κB signaling pathways.

## Discussion

Autophagy is involved in the maintenance of homeostasis in cells by decomposing unnecessary intracellular components[12]. Autophagy-related genes have been identified as disease-susceptibility genes in IBD, and autophagy has been found to play a role in IBD[13–15]. Abnormal granule production and granule secretion into the intestinal lumen by Paneth cells in the small intestine have been observed in mice with decreased Atg16L1 expression[8, 16, 17]. However, in patients with Crohn’s disease, inflammation has been observed in parts of the gastrointestinal tract other than the small intestine, suggesting that the association between autophagy deficiency and inflammation does not depend on only the function of Paneth cells. Here, we hypothesized that autophagy in IECs, an essential biological barrier of the innate immune system in the digestive tract, might be involved in IBD. *Atg*5^flox/flox^/villin-Cre mice showed increased susceptibility to DSS. Moreover, compared to intact IEC-6 cells, IEC6shAtg5 cells showed enhanced expression of proinflammatory cytokines and intracellular ROS accumulation, leading to increased NF-κB p65 phosphorylation via the MAPK signaling pathway.

In our previous study, IEC6shAtg5 cells resulted in intracellular accumulation of ROS[9]. In this study, the expression of proinflammatory cytokines was further enhanced in IEC6shAtg5 cells compared to IEC-6 cells, which was reversed by treatment with an ROS scavenger (NAC). Therefore, when autophagy is suppressed, the accumulated ROS in the cells might fuel a chronic state of inflammation. Because the MAPK/ERK kinase pathway is activated in *Atg5*-silenced cells[9], we suspected that the pathway is associated with the increased levels of proinflammatory cytokines in IEC6shAtg5 cells. We showed that the expression of proinflammatory cytokines in *Atg5*-silenced cells was suppressed by treatment with a MAPK/ERK kinase inhibitor, as expected. The oxidative stress-MAPK axis seems to play pivotal roles in the increase in proinflammatory cytokines in autophagy-deficient enterocytes.

Apoptosis has a complex molecular crosstalk with autophagy[18, 19]. In this study, when IEC6shAtg5 cells were exposed to oxidative stress with lower (10 μM) and higher amounts (100 μM) of H_2_O_2_, the cells exhibited lower viability, accompanied by PARP1 cleavage, an apoptosis marker, than intact IEC-6 cells, indicating that IEC6shAtg5 was more susceptible to oxidative stress-induced apoptosis than intact IEC-6 cells (Fig 3). Apoptosis is also induced by activation of the MAPK signaling pathway through oxidative stress[20–23]. Furthermore, apoptosis in IECs is increased in IBD, resulting in deterioration of the integrity of IECs, the spread of intestinal bacteria, and exacerbation of bowel inflammation[24–27]. Based on these observations, the induction of apoptosis through autophagy suppression may be one of the mechanisms that exacerbate colitis.

In summary, our study revealed that autophagy in IECs plays an essential role in the maintenance of intestinal homeostasis and that autophagy deficiency in cells triggers inflammation through excessive oxidative stress, which promotes NF-κB p65 phosphorylation via the MAPK signaling pathway, resulting in higher expression of proinflammatory cytokines such as IL-1β and IL-6. The development of a new strategy to manipulate autophagy might be beneficial in the treatment of IBD.

## Supporting information

S1 Fig The autophagic flux is inhibited by 3-MA in IEC-6 cells. IEC-6 cells were cultured in a standard culture medium or in a serum-free culture medium without or with 10 mM of 3-MA for 24 h. LC3 expression levels in the IEC-6 cells were determined using Western blot analysis.
(TIF)
