# Supplementary figures and images for: Autophagy deficiency exacerbates colitis through excessive oxidative stress and MAPK signaling pathway activation
Source: PLoS One. 2019 Nov 8;14(11):e0225066. doi: 10.1371/journal.pone.0225066 (PMC6839862; doi:10.1371/journal.pone.0225066)

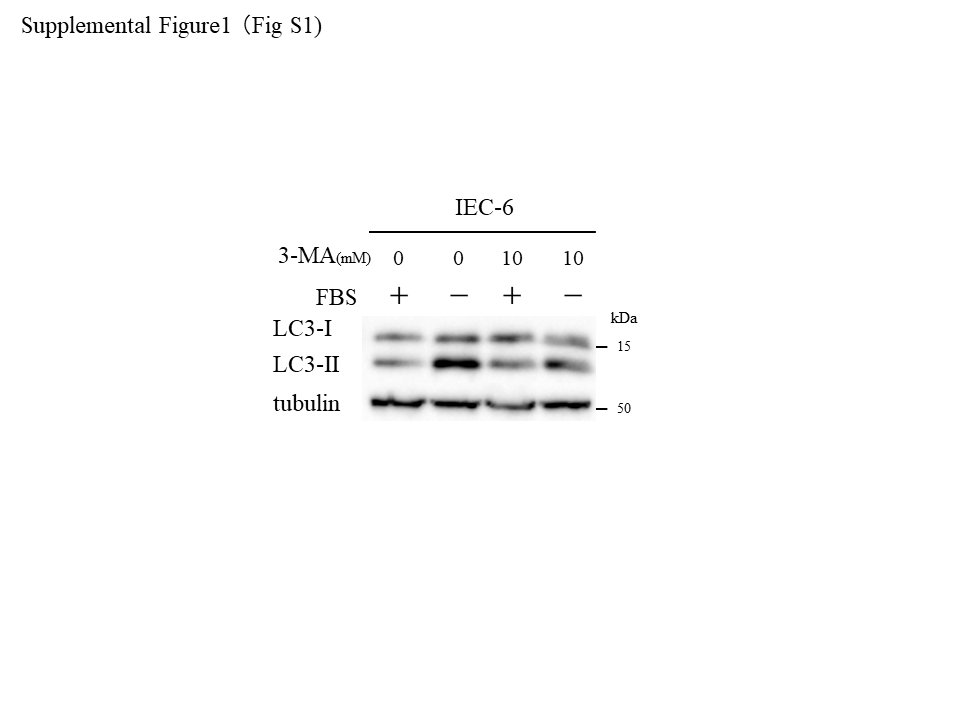

Supplement: S1 Fig — IEC-6 cells were cultured in a standard culture medium or in a serum-free culture medium without or with 10 mM of 3-MA for 24 h. LC3 expression levels in the IEC-6 cells were determined using Western blot analysis. (TIF) [file pone.0225066.s001.tif]
